# Supplementary material for: Evidence supported by Mendelian randomization: impact on inflammatory factors in knee osteoarthritis
Source: Front Med (Lausanne). 2024 May 28;11:1382836. doi: 10.3389/fmed.2024.1382836 (PMC11165061; doi:10.3389/fmed.2024.1382836)
Supplement: Supplementary file 1 [file Data_Sheet_1.ZIP › data/GCST90274785_5e6/785.forest.pdf]

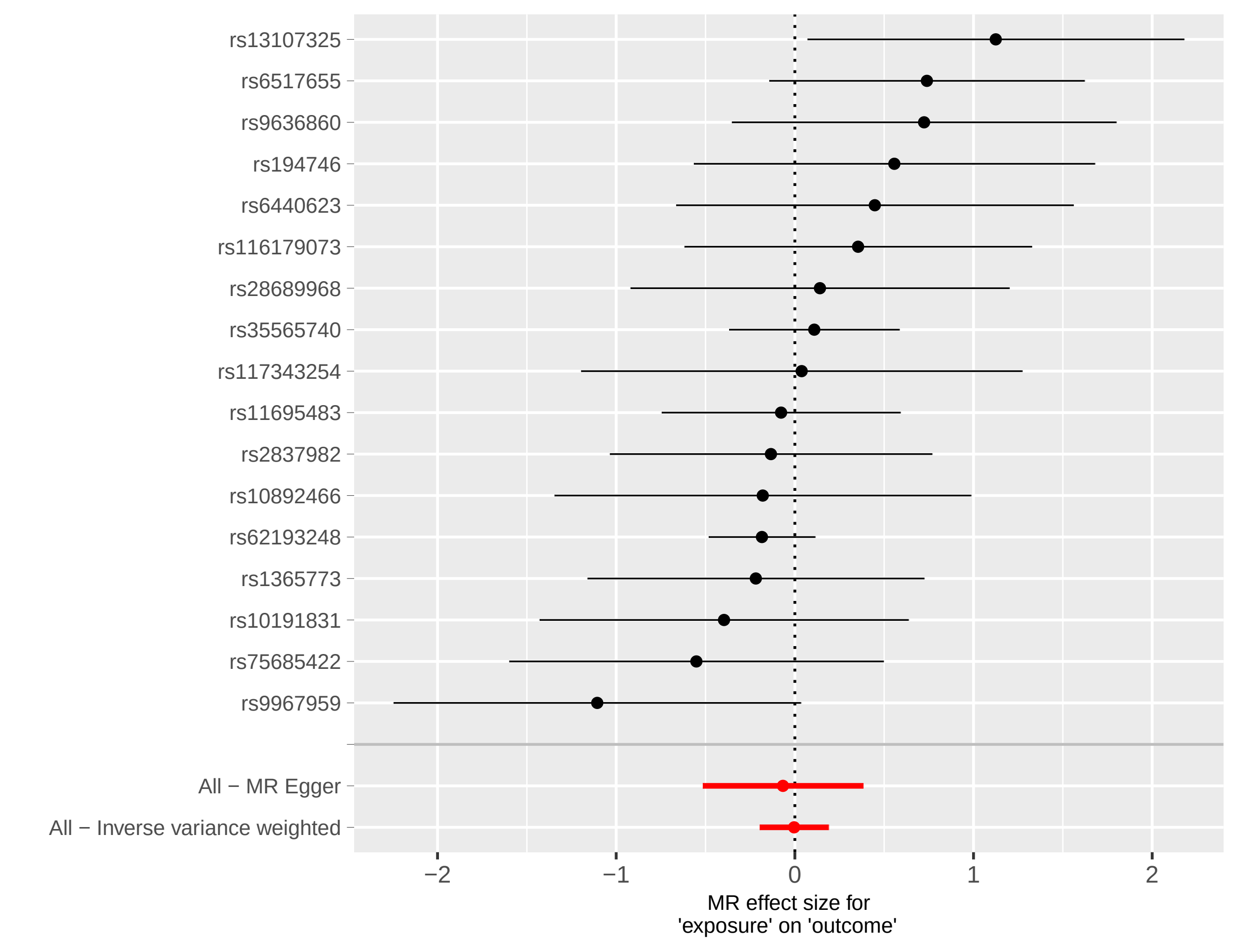
rs13107325
rs6517655
rs9636860
rs194746
rs6440623
rs116179073
rs28689968
rs35565740
rs117343254
rs11695483
rs2837982
rs10892466
rs62193248
rs1365773
rs10191831
rs75685422
rs9967959
All − MR Egger
All − Inverse variance weighted
−2
−1
0
1
2
MR effect size for
'exposure' on 'outcome'
